# Charge Tunable GaAs Quantum Dots in a Photonic n-i-p Diode

**DOI:** 10.3390/nano11102703

**Published:** 2021-10-13

**Authors:** Hans Georg Babin, Julian Ritzmann, Nikolai Bart, Marcel Schmidt, Timo Kruck, Liang Zhai, Matthias C. Löbl, Giang N. Nguyen, Clemens Spinnler, Leonardo Ranasinghe, Richard J. Warburton, Christian Heyn, Andreas D. Wieck, Arne Ludwig

**Affiliations:** 1 Lehrstuhl für Angewandte Festkörperphysik, Ruhr-Universität Bochum, Universitätsstraße 150, DE-44801 Bochum, Germany; julian.ritzmann@ruhr-uni-bochum.de (J.R.); nikolai.bart@ruhr-uni-bochum.de (N.B.); Marcel.Schmidt-a3k@Rub.de (M.S.); Timo.Kruck@ruhr-uni-bochum.de (T.K.); Andreas.Wieck@ruhr-uni-bochum.de (A.D.W.); Arne.Ludwig@rub.de (A.L.); 2 Department of Physics, University of Basel, CH-4056 Basel, Switzerland; liang.zhai@unibas.ch (L.Z.); matthias.loebl@unibas.ch (M.C.L.); giang.nguyen@unibas.ch (G.N.N.); c.spinnler@unibas.ch (C.S.); richard.warburton@unibas.ch (R.J.W.); 3 Center for Hybrid Nanostructures (CHyN), University of Hamburg, DE-22761 Hamburg, Germany; baw2703@uni-hamburg.de (L.R.); heyn@physnet.uni-hamburg.de (C.H.)

**Keywords:** quantum dots, single-photon emitters, quantum photonics, semiconductor epitaxy, growth kinetics

## Abstract

In this submission, we discuss the growth of charge-controllable GaAs quantum dots embedded in an n-i-p diode structure, from the perspective of a molecular beam epitaxy grower. The QDs show no blinking and narrow linewidths. We show that the parameters used led to a bimodal growth mode of QDs resulting from low arsenic surface coverage. We identify one of the modes as that showing good properties found in previous work. As the morphology of the fabricated QDs does not hint at outstanding properties, we attribute the good performance of this sample to the low impurity levels in the matrix material and the ability of n- and p-doped contact regions to stabilize the charge state. We present the challenges met in characterizing the sample with ensemble photoluminescence spectroscopy caused by the photonic structure used. We show two straightforward methods to overcome this hurdle and gain insight into QD emission properties.

## 1. Introduction

Self-assembled quantum dots (QDs) are excellent candidates for creating indistinguishable single-photon emitters [1,2,3,4] as well as entangled photon pairs [5,6]. The most studied and advanced species are InGaAs QDs grown in the Stranski–Krastanov-mode (SK-QDs) in a GaAs matrix. These QDs show excellent electronic and optical properties for quantum applications [4], but their emission wavelength range is limited (typically 1100–1300 nm). Techniques such as indium flushing extend the range to shorter wavelengths [7], but ultimately the range is limited by the bandgap of GaAs (~820 nm @ 4 K). Extending the range to deep-red wavelengths (700–800 nm) allows QDs to fulfill their potential in a more convenient wavelength band. For example, this enables the storage of QD single-photons in quantum memory made of a rubidium atoms ensemble [8] or Silicon vacancies [9], a possibly crucial step for the repeater-based quantum internet. To reach this wavelength range, GaAs QDs in an AlGaAs matrix can be used [6,10,11,12].

Compared to InGaAs SK-QDs, the formation of local droplet-etched (LDE) GaAs-QDs does not rely on lattice-mismatched materials—the strain in the QDs is virtually absent, as no indium is involved [13]. InGaAs QDs have undergone over two decades of developments, whereas the LDE technique is a recent revolution [14]. Some challenges that have been resolved for the established InGaAs dots still exist for the GaAs QDs platform. The predominate one is the noise in semiconductors [15]: GaAs QDs in the bulk tend to suffer from charge noise and charge instability, causing undesired blinking or relatively broad emission linewidths. For InGaAs QDs, these problems are solved by coupling the dots to a Fermi reservoir. For GaAs QDs, despite several attempts over the last decade, charge control has not been established until only recently [16,17]. In the attempt from Zhai et al. [1], the GaAs QDs can be deterministically charged from an electron reservoir over a tunnel barrier, supported by the Coulomb blockade. The key is the engagement of an n-i-p diode structure. Charge noise and instability are suppressed, and the GaAs QDs are blinking free. The optical linewidths of such QDs are, therefore, as narrow as the lifetime-limit.

It is intriguing to study these GaAs QDs in the n-i-p diode in more detail, especially from a sample growth perspective. We note that, as is pointed out by Zhai et al. [1], most of the QDs emitting at wavelengths below ~785 nm show excellent optical properties, i.e., 7 out of 10 randomly chosen QDs have a linewidth narrower than 1.2 times the lifetime-limit. However, for QDs emitting above ~785 nm, the resonance fluorescence is difficult to measure. In this paper, we discuss the molecular beam epitaxy (MBE) growth of the sample used in Ref. [1] in detail. We emphasize our efforts to reduce the build-in of impurities to suppress charge noise. As we extended the effort on the LDE process, we also evaluate the effect of the used parameters for the grown QDs. Under the utilized growth condition, we identify a bimodal QD distribution. Such a bimodal distribution is related to the wavelength-dependent optical properties of the low-noise GaAs QDs mentioned above [1]. The results extend to GaAs QDs used in other publications, as similar growth parameters were used [18,19].

Further, we link the previous findings with ensemble photoluminescence (PL) measurements. We address challenges in characterizing QDs embedded in planar-cavity structures by PL spectroscopy. We find that the cavity structures strongly distort the ensemble PL. We present simple ways to quickly determine if a spectral change is induced by the planar-cavity heterostructure or a change in QD properties and how an undisturbed spectrum can be obtained by cleaved-edge PL, that is, PL recorded from the side facet of the cleaved wafer.

## 2. Materials and Methods

### 2.1. Sample Growth

**Figure 1:**
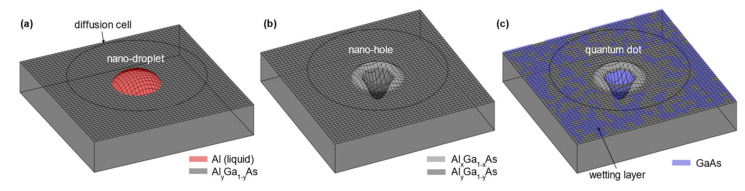
Schematic illustration of QD fabrication by LDE and hole filling. (**a**) Aluminum droplets form due to sufficiently low As-flux and etch into the surface. (**b**) A nano-hole is left behind after the etching process is terminated, and etched material agglomerates in a wall surrounding the hole. In the case of aluminum used for etching, the wall has a higher Al concentration then the matrix material [28]. (**c**) GaAs is deposited and fills the nanohole. Nearby material diffuses into the hole, and the diffusion area (Voronoi-cell) correlates with the filling level [29]. In between the nanoholes, GaAs forms a quantum well, which is called a wetting layer, in accordance with the term used for InGaAs QDs.

To achieve sufficient purity that is needed for quantum applications, MBE is a good choice, as it is known to yield material with excellent purity levels [20,21,22]. A technique to form GaAs QDs in an MBE setup is the infilling of self-assembled nanoholes formed by LDE [13,23]. The usual stochiometric ratio group III-V arsenic (As)-based compound semiconductor growth in MBE uses an As-overpressure (i.e., the group V to III ratio is much larger than one) [24] to form layers, wires, and QDs of GaAs, AlAs, InAs, or the corresponding alloys. In LDE, a Group III element (e.g., aluminum) is deposited while the As-pressure is strongly reduced [14,25]. This element forms nanodroplets on the surface in Volmer–Weber mode (Figure 1a) [26]. With a small flux of arsenic, the droplets etch into the underlying material (e.g., AlGaAs) and form nano-holes (Figure 1b) [26,27]. As-flux is restored up and GaAs is deposited, which diffuses into the holes. Thereafter, an AlGaAs capping layer is deposited, and the GaAs-filled hole forms a 3D energy confinement, also known as a QD (Figure 1c).

All sample growth was performed on a modified Riber Epineat III/V S solid-source MBE system (Bochum, Germany). Undoped full 3” GaAs wafers with (001)-orientation (miscut < 0.1° as specified by the vendor) were used. The temperatures were calibrated before growth by pyrometer measurements. All samples were deoxidized at roughly 650 °C under an As-beam-equivalent pressure (BEP) of 9.6 × 10^−6^ Torr. An As-valved-cracker cell was used, with the cracking zone operating at 700 °C. To achieve high-sample-quality and low-noise QDs, it is crucial to keep the impurity levels as low as possible, especially in layers close to the QDs or with a high Al percentage. The strategy for reducing impurities in the relevant structure was to use a high substrate temperature, low As-flux, and short growth interrupts. A compact overview of the sample and the important growth parameters is given in Appendix A (Table A1).

Sample growth is exemplified in the structure used by Zhai et al. [1]. In the discussion, this sample is referred to as the “photonic sample”. Layer growth was performed under an As-BEP of 9.6 × 10^−6^ Torr and at 620 °C. On the deoxidized substrate, a 100 nm GaAs buffer layer was grown in 10 nm steps with intermediate annealing breaks. On top, 22 layer pairs of a 2.8/2.8 nm GaAs/AlAs short-period superlattice (SPS) were deposited. This was followed by 10 pairs of an Al_0.33_Ga_0.67_As/AlAs distributed Bragg reflector (DBR), designed for a 795 nm reflection maximum (stopband-center) at 4 K.

After that, the back-contact region was fabricated. All doped layers and their buffers consisted of Al_0.15_Ga_0.85_As to suppress DX centers energetically located below the conduction band edge [30]. A 50 nm intrinsic Al_0.15_Ga_0.85_As layer was deposited followed by a 150 nm n-doped Al_15_GaAs back contact. The intended doping concentration was 2 × 10^18^ cm^−3^. To reduce Si-surface segregation, the temperature was decreased to 605 °C and a 5 nm Al_15_GaAs layer was grown. The temperature was increased to 620 °C and an additional 15 nm Al_15_GaAs tunnel barrier was deposited. The aluminum cell was adjusted for a 33% Al concentration to fabricate the 10 nm Al_0.33_Ga_0.67_As matrix.

On the matrix, LDE was performed, started by closing the As-cracker valve. To reduce impurities, the As-pumping time was kept relatively short (240 s). During pumping, the substrate temperature was driven up to 635 °C. For etching, 0.09 nm Al was deposited with the addition of 1 ML equivalent (ML_eq_) to counter As-surface termination, resulting in a total amount of 1.3 ML_eq_. The growth rate equivalent was 0.1 nm/s. Etching was carried out for 60 s, and As-BEP is estimated in the range of 6–9 × 10^−8^ Torr. The As-valve was opened again to match a BEP of 9.6 × 10^−6^ Torr and the surface was annealed for 60 s. The etched holes were filled with 2 nm GaAs with a growth rate of 0.2 nm/s. This amount, in combination with the used etching parameters, yields QDs emitting in the desired wavelength range (>780 nm). An annealing break of 120 s followed, in order to allow the GaAs to diffuse into the holes. The formed QDs were overgrown with 273.6 nm Al_0.33_Ga_0.67_As. Thereafter, the p-doped epitaxially grown gate was fabricated. The epi-gate contains a 65 nm carbon-doped Al_0.15_Ga_0.85_As layer. The targeted doping concentration was 2 × 10^18^ cm^−3^. The sample was finished with a 10 nm Al_0.15_Ga_0.85_As layer and a 5 nm GaAs oxidation protection cap layer, both with an aimed doping concentration of 8 × 10^18^ cm^−3^.

The emission wavelength of LDE GaAs QDs can be adjusted by the amount of deposited GaAs after etching [10]. To achieve QD emission in the desired wavelength range, simplified test samples were grown. There are two slight modifications in the test samples. The main difference is the lack of a DBR and a p-doped top gate. Both changes reduce growth time and make it possible to achieve an undistorted QD PL spectrum without the influence of the photonic structure. Another change was the lower LDE temperature of 630 °C. The test sample with 2 nm GaAs filling was showing a QD peak in the targeted wavelength region of 795 nm at 4 K. The underlying reason is to match the QD emission wavelength with the rubidium quantum memory that is readily available [8]. It is used as a reference for the photonic sample and is denoted by the absence of a DBR as the “non-photonic sample”.

Furthermore, to gain insight into the structural properties of the GaAs QDs, additional samples were prepared for atomic force microscopy (AFM) measurements. In these samples, an additional LDE process was involved at the sample surface. This LDE process was, however, terminated shortly after the hole etching, allowing direct access to the shape of the nano-holes. Finally, samples with a reduced Al-etch amount were grown. The amount was reduced until no QDs could be detected by PL. The Al was deposited with stopped rotation, which creates an Al-material gradient. Through this, the effective Al quantity available for droplet formation can be estimated. A detailed explanation is attached in Appendix B.

### 2.2. Characterization

AFM measurements were performed with a Veeco Dimension 3200 (Hamburg, Germany). For density measurements, a large scan covering an area of 50 × 50 µm^2^ was conducted. To obtain a better view of the morphology of the surface and the etched holes, additional higher-resolution scans were conducted.

To measure the QD density on the photonic sample, µ-PL measurements were performed at 4 K. The µ-PL contains the spatial and spectral information of the QDs in a relatively small range—one µ-PL mapped out an area of 24 × 24 µm^2^ on the sample. The µ-PL was excited with a narrow-band He-Ne laser (632.8 nm) and resolved with a spectrometer. The spectrometer has a measurement range from 767 to 810 nm, which covers the emission wavelengths of most QDs. A bias voltage of −0.4 V was applied for charge state stabilization.

**Figure 2:**
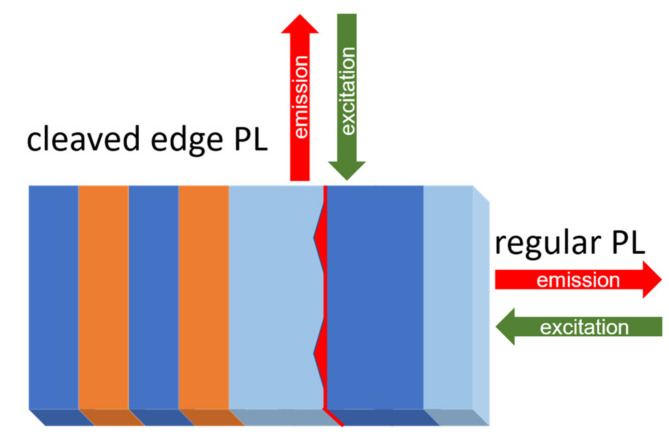
A schematic illustration of regular and cleaved-edge PL. While in regular PL, light emitted from the top of the layer structure is measured, cleaved-edge PL measures the light emitting from the side.

Ensemble PL, which mapped out the ensemble spectrum of the whole wafer, was performed at a temperature of 100 K. The wafers were clamped onto a copper plate, which was glued onto a stainless-steel liquid nitrogen reservoir. A 532 nm laser was used for excitation and a wide-range (340–1020 nm) compact spectrometer was used for light detection. The laser spot diameter was approximately 100 µm. Various excitation powers were used depending on the response of the sample. To study the influence of the photonic features, 0.5 mm-thick pieces from the photonic and non-photonic samples were cleaved and measured from the side, which is referred to as “cleaved-edge PL”. The corresponding excitation and detection schemes are presented in Figure 2.

The reflectance of the photonic sample was measured using a reflectometer. The measurement took place at room temperature. The device uses a white light source that is focused on the sample, and the spot diameter is roughly 4 mm. The light reflects onto a motorized optical grating and is detected by a coupled Si-InGaAs detector, toggling at 1000 nm. The recorded spectrum is then transformed into the reflectance spectrum.

## 3. Results and Discussion

### 3.1. Evaluation of the Growth Parameters

Subsequent reduction of the Al for etching showed that the formation of light-emitting QDs with the set growth parameters already occurs at a deposited amount of roughly 0.4 ML_eq_. Further details on the determination are shown in Appendix B. This shows that for our parameters of a 635 °C growth temperature, 6 × 10^−8^ < As-BEP < 9 × 10^−8^ Torr, and 240 s pumping time, the Al_0.33_Ga_0.67_As surface is not completely As-terminated. The use of 1.3 ML_eq_ Al, as with the photonic sample, results in an effective amount of etching material, of at least 0.9 ML_eq_, which is at least a factor of 3 higher than intended. If the etch amount exceeds a certain threshold, which is dependent on the As-BEP and the growth temperature, a second nucleation phase of the metal droplets occurs, which leads to a bimodal size distribution [31,32]. A bimodal QD distribution could explain the findings of two groups of QDs [1]: One group of QDs has narrow linewidths, while the other has broad linewidths.

**Figure 3:**
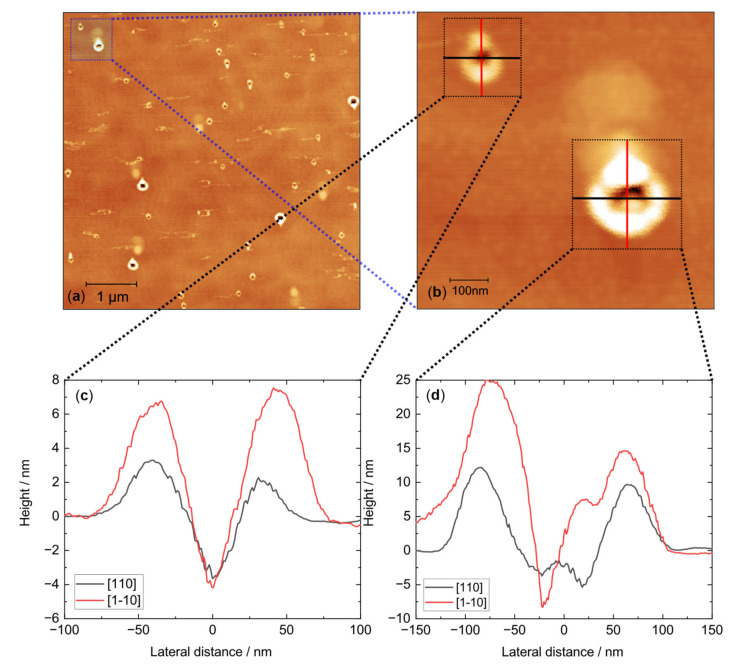
AFM images of the etched nanoholes. (**a**) An overview of the sample surface. A bimodal hole distribution is present, as well as traces of droplet running. (**b**) Representative detailed image of a deep hole (**bottom right**) and a shallow hole (**top left**). (**c**) Height profile of the shallow hole seen in Figure 3b. The shallow holes are 4–5 nm deep, with higher, but not perfect, symmetry. (**d**) Height profile of the deep hole seen in Figure 3b. The deep hole mode etched approximately 10 nm deep with strong asymmetry in the different crystal orientations.

AFM investigations are a well-suited tool to detect a bimodal distribution and to assess its properties. In Figure 3a, an overview AFM image of the LDE sample with etched holes on the surface is presented. It clearly shows a bimodal hole distribution with low density, deep holes, and higher-density shallow holes. The surface also shows signs of droplet running [33]. The results are a clear indication that the Al/As-ratio used resulted in a bimodal hole distribution [31,32]. This matches well with the quantification of the effective etch material, which finds at least three times more etch material was used than intended.

In Figure 3b, a representative detailed image of the two modes is presented. Representative height profiles are plotted in Figure 3c for the shallow hole mode with a depth of approximately 4–5 nm and in Figure 3d for the deep hole mode with a depth of approximately 9–13 nm (ensemble range). The deep holes show strong asymmetry with an almost squared shape. The shallow holes appear round with a slight asymmetry depending on the crystal orientation. It is noted that the shallow holes show a wide relative size distribution with very small and slightly larger holes, which can be seen in Figure 3a.

The AFM-measured density of the deep holes is (0.13 ± 0.01) µm^−2^, while the shallow hole density is (0.64 ± 0.12) µm^−2^. Additional µ-PL mapping of the QDs on the photonic sample shows a total density of (0.39 ± 0.01) µm^−2^ in the emission range of 767 nm to 810 nm (4 K temperature). An exemplary µ-PL map is attached in Appendix C. The total density is only half the value determined by AFM. This is, though, understandable as not all QDs are expected to be optically active, and even if they are all optically active, we cannot assume all the QDs emit in the measured region of the µ-PL. The smallest QDs emit at shorter wavelengths, which are cut off by the limited spectrometer range (lower limit: 767 nm). The short wavelength extension of the QD emission can be seen in the following ensemble PL section.

To link these findings to the previous single QD experiments, they are summarized briefly. The study showed that out of 10 QDs emitting under 785 nm, 7 showed close to lifetime-limited linewidths (<20% broader than lifetime limit). The GaAs QDs were charge-tunable by the Stark effect. They possessed stable emissions without telegraph noise, owing to the low impurity levels during the growth and a successful demonstration of the diode structure. Additionally, it was observed that QDs emitting above 785 nm were less optimal in terms of optical properties [1]. The bimodal hole distribution explains these findings: The deep mode is strongly asymmetric, which leads to a large fine-structure splitting (FSS) [23]. The shallow holes can be assigned to the QDs with good photonic properties. Due to their smaller height, they emit at shorter wavelengths. The higher symmetry causes a smaller FSS [23,34]. The broad size distribution causes only a certain amount (“7 out of 10”) of QDs to show close to lifetime-limited linewidths.

The FSS achieved is remarkable in comparison with previous studies. Huo et al. were able to achieve near-perfectly symmetric etched holes. The QDs obtained by filling these highly symmetric holes showed an FSS on the level of 4 µeV [23]. Our shallow holes lack this high grade of symmetry as seen in Figure 3b, but 3 out of 10 QDs also showed an FSS of roughly 4 µeV with 5 others still below 10 µeV [1]. It should be noted that Huo et al. determined the FSS using non-resonant excitation (polarization-dependent PL) [23], while Zhai et al. used resonant excitation [1]; however, an effect of the excitation method on the FSS is not expected. A plausible explanation for the low FSS is the high ensemble inhomogeneity of shallow holes, which also contain a certain fraction of highly symmetric holes. Another possibility is that the GaAs fills only the lower part of the shallow holes, which could be enhanced by the relatively high walls left by the etching process. As can be seen in Figure 3b, the lower region of the holes is highly symmetric, which could lead to a low FSS. Unfortunately, confirmation using AFM-measured filled holes is unachievable due to ensemble inhomogeneity, as it is impossible to find two identically etched holes to determine the morphology from the difference in depth profiles.

Resonant fluorescence measurements on one of the QDs showed a sharp linewidth of 0.64 GHz, which is very close to the determined lifetime limit of 0.59 GHz [1]. It can be concluded that the very low noise level indicates a QD vicinity containing low impurity levels. Heyn et al. recently correlated the trap density of the environment with the Stark-effect-generated charge noise [35], which compromises QD emission and broadens the emission lines. The low charge trap density also contributed to the suppression of blinking, the absence of which was also demonstrated by Zhai et al. [1].

### 3.2. Ensemble Photoluminescene

**Figure 4:**
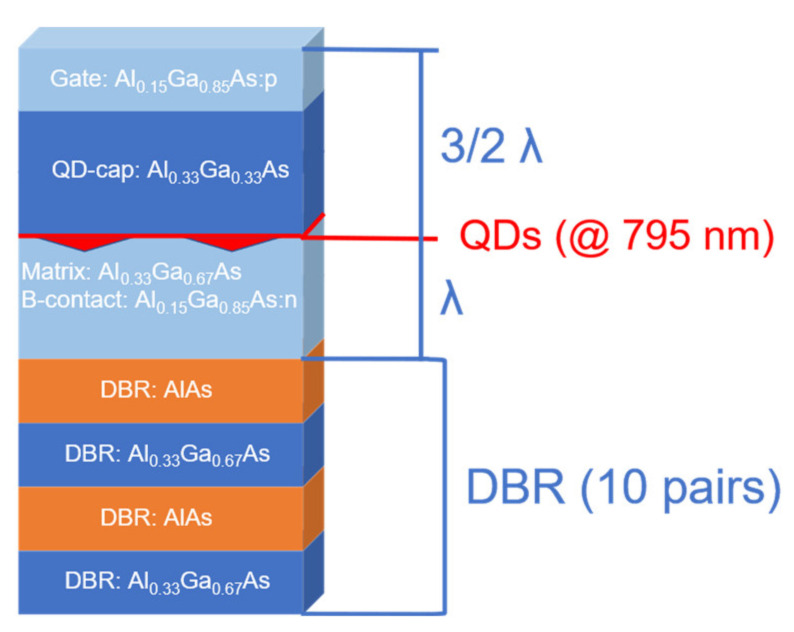
Design schematic of the photonic sample. Color scheme: Orange is AlAs, deep blue is Al_0.33_Ga_0.67_As, and light blue is Al_0.15_Ga_0.85_As. The QD material is GaAs (red). The 10 nm Al_0.33_Ga_0.67_As matrix is neglected.

To enhance photon coupling, QDs are often embedded into photonic structures [36]. This is also applied for the photonic sample, which is coupled with a 10-layer-pair DBR below the QDs. The equivalent vertical distance from the QDs to the DBR is designed as λ and to the surface as λ 3/2 (@ 795 nm over the according refractive index *n*), as schematically illustrated in Figure 4. This has two main effects, the first one being the formation of photonic cavity, which can be proved by measuring the reflection spectrum. The second effect is the lateral positioning of the QDs in the maximum standing wavefield, which additionally enhances the QDs emission over other features. Thereby, induced effects on the according ensemble PL emission are investigated in the following chapter.

**Figure 5:**
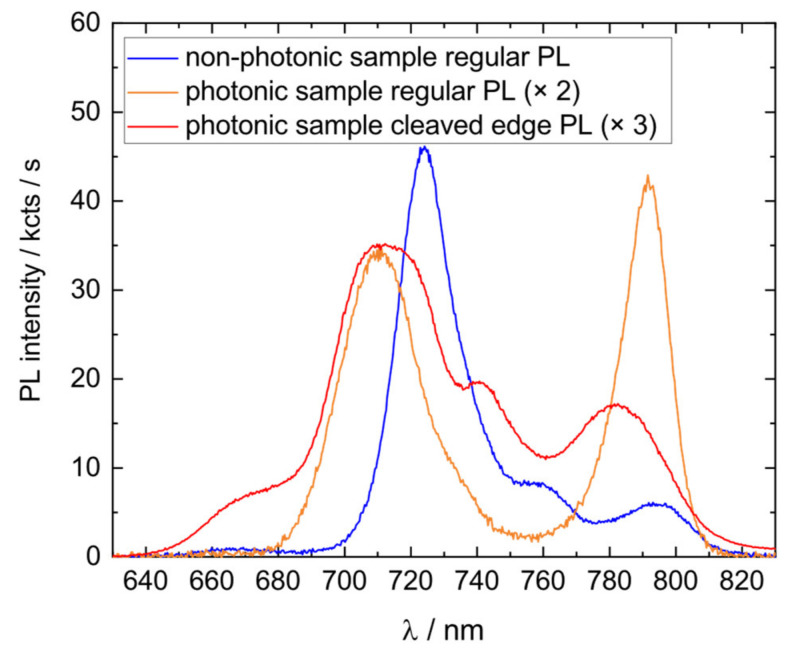
Comparison of regular ensemble PL emission of the photonic sample (orange line), non-photonic sample (blue line), and cleaved-edge PL of the photonic sample (red line). For better comparability, multiple factors were used.

In Figure 5, ensemble PL spectra from the center of the wafer are displayed. The PL spectrum of the photonic sample is plotted (orange line) in comparison to the pre-grown non-photonic sample (blue line), which contains QDs fabricated with mostly the same parameters. The non-photonic sample has no DBR and no emission-matched layer thickness, so the above-described effects should be absent. Spectral peaks at 710 and 725 nm can be assigned to the wetting layer (~710 nm) or Al_15_GaAs (~725 nm) emission. The non-photonic sample shows another peak at 762 nm, which can be correlated to the second QD mode, which results from a bimodal hole distribution. The main QD peak for the photonic sample is centered at 791 nm and for the non-photonic sample at 797 nm. The peak is much sharper on the photonic sample and appears more prominent when compared to the other PL features, especially in comparison to the Al_0.15_Ga_0.85_As peak. Such a dramatic change could naively be attributed to the assumption that the QD ensemble was strongly homogenized and enhanced by different growth conditions, for example a longer growth and therefore change of As-etch-BEP, or comparable changes.

However, investigations showed this was not the case for the photonic sample; instead, the real cause of the spectral change can be assigned to the layer design distorting the photon outcoupling and thus PL emission. The cavity structure rejects light outcoupling for certain wavelength ranges and enhances light outcoupling near the stopband cavity dip. The PL-spectrum is therefore enhanced and sharpened at the QD emission peak, and other peaks such as the Al_0.15_Ga_0.85_As are suppressed. To neutralize the cavity effect and obtain a clear PL spectrum, cleaved-edge PL was performed on the photonic sample, which can be seen in Figure 5 (red line). As a proof-of-concept, this was also performed on the non-photonic sample and is attached in Appendix D, where no significant change in the emission spectrum can be seen. However, for the photonic sample, the change is significant. The QD peak appears much broader and less prominent compared to the regular PL measurement. The photonic sample also shows a feature at 745 nm, which can only be suspected in the right shoulder of the Al_0.15_Ga_0.85_As peak of the non-photonic sample. This peak is ascribed to the 5 nm GaAs antioxidation layer on top of the samples, resembling a quantum-well-like structure.

The QD peak of the photonic sample extends to much shorter wavelengths compared to the non-photonic sample; the second mode peak does not appear separately. It can be assumed that this peak is slightly red-shifted in the photonic sample and merged with the main QD peak. We attribute this change to slightly different growth parameters in the photonic sample (5 °C hotter). A higher temperature causes the droplets to etch slightly deeper, which forms red-shifted QDs. The relatively strong change is caused by the second mode being “shallow-holes”. Due to their small size, the effect of this magnitude could most likely be seen in shallow-mode QDs.

**Figure 6:**
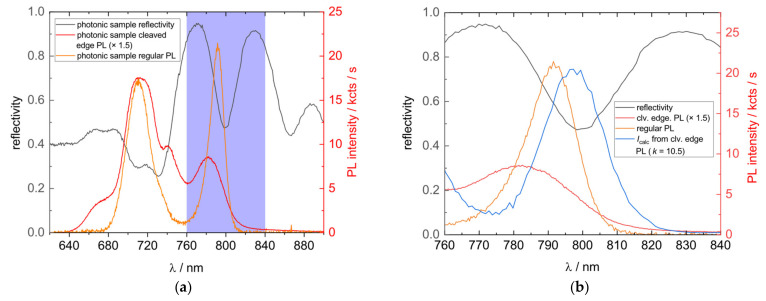
(**a**) Comparison of the measured reflectivity (black line), regular (orange line), and cleaved-edge PL (red line) of the photonic sample. For better comparability, a multiplication factor was used at the cleaved-edge PL spectrum. (**b**) Magnified area around the QD peak. The calculated PL spectrum (*I*_calc_), obtained by combination of the cleaved-edge PL and reflectivity spectrum is additionally plotted (blue line). The *k* value used is 10.5. PL measurements were performed at 100 K, while the reflectance was measured at room temperature.

As already indicated above, we find the origin of the different PL-spectra for normal and cleaved-edge PL in the photonic structure. With great evidence, this effect can be seen in the dip of the reflection spectrum, which has been measured and displayed for the photonic sample in Figure 6 (black line). Alongside this, the cleaved-edge PL spectrum (orange line) is plotted, which can be assumed as an undistorted spectrum. The lower the reflectivity near the stopband cavity dip, the more photons can escape. This distorts and redshifts the position of the QD peak by roughly 10 nm as well as strongly cutting off the blue side of the peak. Other features are suppressed, leading to a higher prominence of the QD peak in the total spectrum. The prominence is further enhanced by the QDs being placed in the maximum standing vacuum wave field, which is shown in Appendix E, providing additional amplification of QD emissions over other light-emitting features. Interestingly, a very simple approach is found to reassemble the photonic sample QD emission spectrally near the cavity dip by combining the reflectance measurement with the cleaved-edge PL:

(1) $$I_{\mathrm{calc}}\left(\lambda \right)=\;k\cdot I_{\mathrm{CE}}\left(\lambda \right)\;\left(1-\;R\left(\lambda \right)\right)$$

The undistorted cleaved-edge intensity $I_{\mathrm{CE}}$ is multiplied by one minus the measured reflectivity R to account for the wavelength-dependent PL distortion of the cavity. The empirical factor k accounts for an intensity enhancement through the cavity and standing wave field as well as the different measurement conditions. In Figure 6b, the model is applied and plotted (blue line). It reshapes the PL peak measured by regular PL quite well, but a redshift from 791 nm to 797 nm is still visible as well as a slightly broader peak. The reason for this could be the spectral resolution of the reflectometer and the reflectance spectrum being measured at room temperature while PL was performed at 100 K. Simulations show that the cavity-induced stop-band dip is shifted by 6–8 nm upon cooling the sample from 300 to 100 K, which fits well with the observed difference. A reflectivity measurement at 100 K is expected to enhance the agreement but was not technically possible.

**Figure 7:**
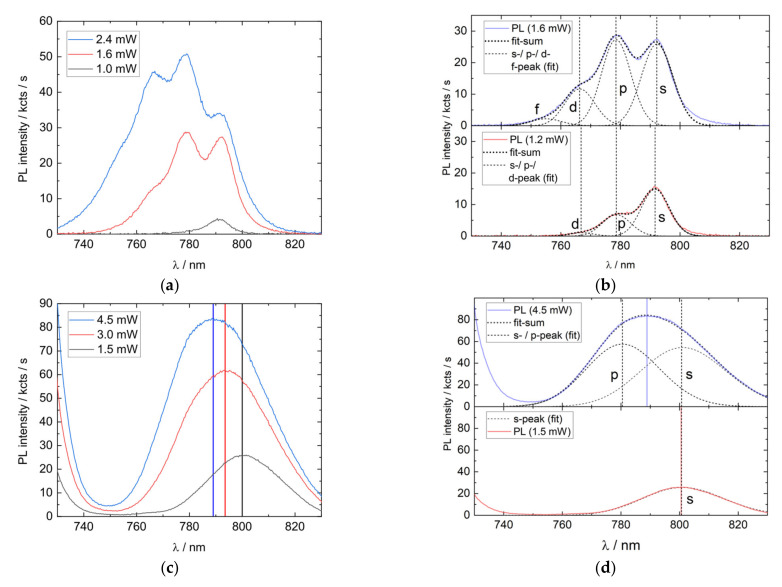

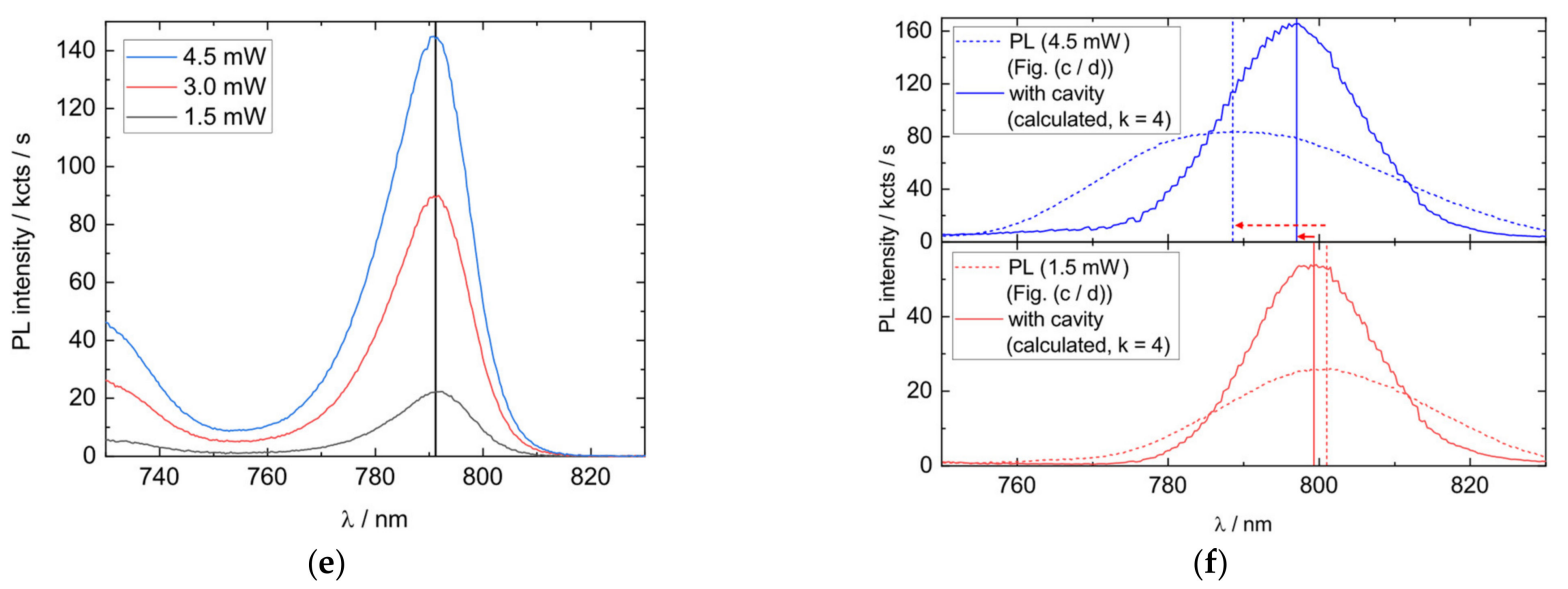
PL power series measurements and corresponding illustrations. (**a**) GaAs QDs with a narrow ensemble and separatable ground-state and excited peaks. (**b**) Illustration of the narrow ensemble structure by fitting Gaussian peaks, representing s-, p-, d- and f-states. The single-peak FWHM for the energy-states is in the range of 20 meV to 27 meV. (**c**) PL power series of GaAs QDs inseparable energy states caused by a broader ensemble. Nevertheless, higher excitation power shifts the peak maximum to shorter wavelengths, which is caused by shell filling and thus brighter excited peaks. (**d**). Illustration of the spectral composition in (**c**), by fitting Gaussian peaks to s- and p-states. The ensemble FHWM is in the range of 60 meV to 66 meV. (**e**) PL power series with cavity dominated spectrum. The PL peak shows no significant position change with higher excitation, as the cavity dip does not respond to higher excitation but strongly influences PL emission. (**f**) Illustration of the spectral change in (**e**) by combining the PL-spectrum from (**c**) with empirical Equation (1), where the measured reflection spectrum of the photonic sample is used (comp. Figure 6b). The peaks are narrowed and the peak position shifts by changing the laser excitation power only 19% compared to the raw PL spectral change. Vertical lines serve as a guide-to-the-eye, highlighting the position of peak maxima.

Another method to differentiate between an ensemble and a cavity-enhanced peak without modifying the sample is to conduct a power series PL measurement. When performing a power series on QDs, shell filling sets in for increased excitation power and higher states become excited, emitting photons at shorter wavelengths, which reveals additional QD properties. The first measurement (Figure 7a) shows a sample with a narrow ensemble, where energy peaks can be well separated. The second measurement (Figure 7c) was performed on another sample, which hosts a broad ensemble caused by different growth parameters. The energy peaks cannot be separated easily and only a broad PL peak appears. The third measurement (Figure 7e) shows QD emission from a broad ensemble, which is distorted by a photonic structure; in this case, the previously discussed photonic sample.

The right side (Figure 7b,d,f) provides an idea of the PL spectral change due to increased excitation. For this purpose, Gaussian peaks are fitted in Figure 7b,d, symbolizing the ground state (s-state) and excited states (p-, d-, and f-states). The value of the peak width (full width at half maximum, FWHM) divided by the peak distance determines the shape of the summed spectrum and is a measure for peak visibility. For Figure 7b, this value is (~0.8 to 0.9 (s to p)) and for Figure 7d, it is (~1.5 to 1.6 (s to p)). The detailed-fit parameters are shown in Appendix F. Figure 7f illustrates the cavity effect, using the measured PL spectra from Figure 7c,d and combining it with the cavity dip of the photonic sample (Figure 6), simply by applying Equation (1). For demonstration, the empirical *k* factor is arbitrarily chosen to be 4 for both spectra. The k-factor differs from the previous value (10.5), because in this case, a normal PL measurement instead of cleaved-edge PL was used. The increased intensity is only present due to the increase of the brighter “raw” PL-spectrum. The small observed shift of the calculated PL-spectrum is caused by the raw PL signal (s-state: ~801 nm) not being perfectly matched with the cavity (center: ~797 nm). The effects shown can presumably also occur in other spectral ranges, e.g., with InGaAs QDs.

The narrow ensemble in Figure 7a is easily understandable. At low power, only the ground state becomes excited (~792 nm). With increasing excitation, more shells are filled and two additional excited peaks become visible, located at (~779 nm) and (~766 nm). The distinguishability is possible due to the FWHM being smaller or in the same range as the energy-state separation, as illustrated in Figure 7b. For the broad ensemble presented in Figure 7c, state filling also occurs with higher excitation, but due to the strong inhomogeneity, only a broad peak appears with no visible single energy state emission. The state filling can nevertheless be observed by the blue shift of the peak maximum. The explanation is illustrated in Figure 7d, where the FWHM of the excited states is much larger than the energy splitting, resulting in an overall peak with no outstanding energy features. When excited states gain higher occupation, they also have a higher contribution to the overall signal, which results in a blue shift.

As in Figure 7c, the measurement in Figure 7e also shows only one QD-related emission peak. The peak appears sharper, but a situation such as the one shown in Figure 7d could still apply, assuming a simultaneous reduction in FWHM and energy splitting. The crucial difference consists of the absence of a peak-shift to shorter wavelengths, as found in the previous case. The excitation power increases, more shells are excited, but the maximum stays at 791 nm. The spectrum is therefore dominated by a cavity effect. Figure 7f illustrates the effect, the broad QD emission shows a blue shift, but due to the non-excitation-dependent cavity outcoupling, the PL peak stays at its almost fixed position, with a small shift caused by a cavity/QD mismatch. This finding can be used to differentiate, within minutes, whether a PL peak is shaped by the ensemble or the cavity, and prevent misjudgments regarding QD growth onto and into photonic dielectric heterostructures. A more thorough model will take the coupling to a charge reservoir [37] and the standing wave field amplitude into account. If the PL is found cavity-dominated and further investigation is desired, the sample can be subsequently characterized by cleaved-edge PL.

## 4. Conclusions

To conclude our findings, we showed that the used growth parameters led to a bimodal QD distribution with deep, strongly asymmetric, and shallow holes with a higher yet not perfect symmetry. It is highly likely that the shallow holes are the species showing excellent properties in the previous work [1].

These findings provide a good perspective for further improvements by increasing the symmetry of the dots as well as growing only a single and uniform QD mode. Better symmetry leads to a lower and thus preferred FSS, and the growth of uniform QDs with a slightly lower density would be beneficial to reduce the charge noise from QDs nearby. This can be achieved by (a combination of) etching under higher As-flux conditions [32], by using lower temperature [38] or less etch material [23]. These optimizations nevertheless should be conducted carefully while keeping the impurity levels in the matrix material low, as some of the practical solutions might be a tradeoff that will increase impurities (e.g., lower temperature). With a further optimized LDE process, we expect to exceed the already-good properties that the photonic sample showed, enhancing, for example, the spin properties. Nevertheless, the possibility to fabricate high-quality QDs by growing a bimodal distribution should be noted. However, the generally broader ensemble should be considered, as well as the higher susceptibility to changes of growth parameters such as As-BEP, pumping time, or growth temperature.

The efforts to reduce the charge trap density in the QD environment and gain charge control by utilizing an n-i-p-diode structure have resulted in significant improvements, such as low charge noise and suppression of QD blinking. We expect to be able to decrease the impurity density even further, as there is still tolerance for shortening growth breaks where impurities can accumulate. In addition, matrix material with less aluminum content could be used, which would reduce the binding probability for impurities.

In the second part, a strong change of the QD ensemble PL spectrum by the surrounding heterostructure was shown, which could be mistaken for homogenization of the ensemble. It was demonstrated that the change is induced by the photonic properties of the enclosing cavity structure, by means of correlating PL spectra with reflectance measurements. We also presented how cleaved-edge PL can be used to record an undistorted spectrum and how a power series can be utilized to distinguish between a PL peak shaped by a cavity structure and an inhomogeneously broadened QD ensemble sample with overlapping excited states.

## Figures and Tables

**Figure A1:**
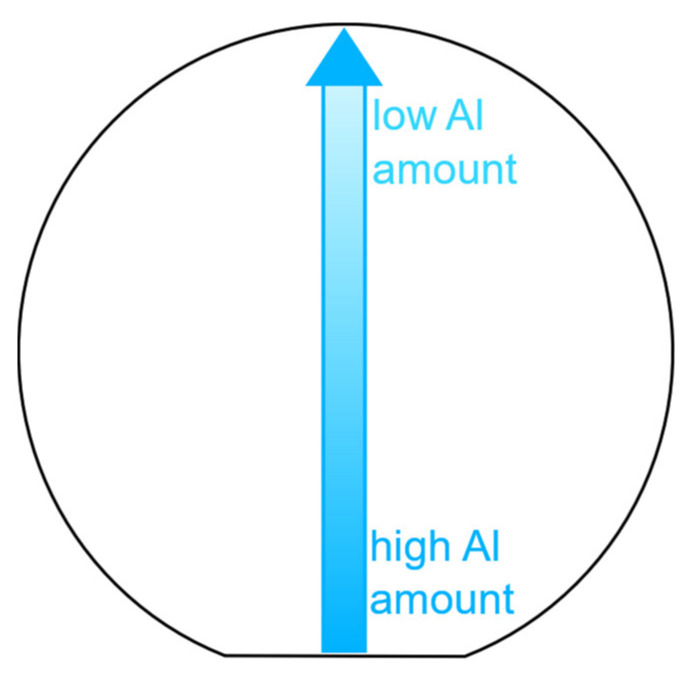
Schematic illustration of the gradient growth technique. The Al-cell points to the bf.

**Figure A2:**
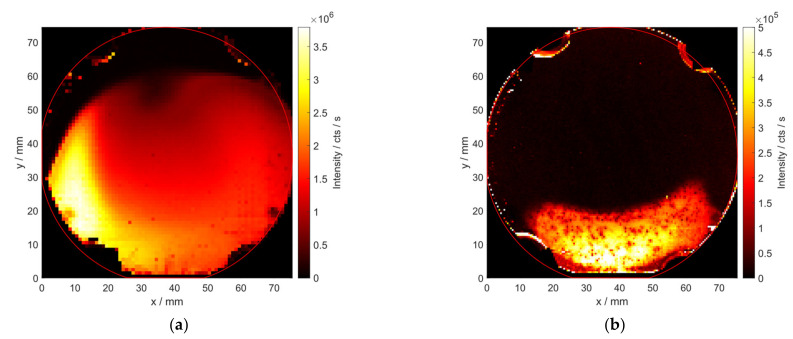
Integrated PL intensity maps (750–830 nm) of samples with low Al etch material. (**a**) 0.5 ML_eq_ central Al shows a convex-shaped transition near 60 mm. (**b**) 0.3 ML_eq_ central Al shows a concave-shaped transition near 20 mm.

**Figure A3:**
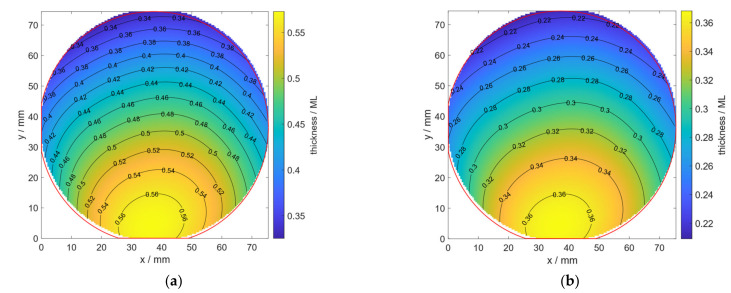
Simulated amount of Al with (**a**) 0.5 ML_eq_ and (**b**) 0.3 ML_eq_ thickness. The simulation is based on gradient growth and PL mapping of quantum wells, from which the effusion cell profiles are calibrated.

**Figure A4:**
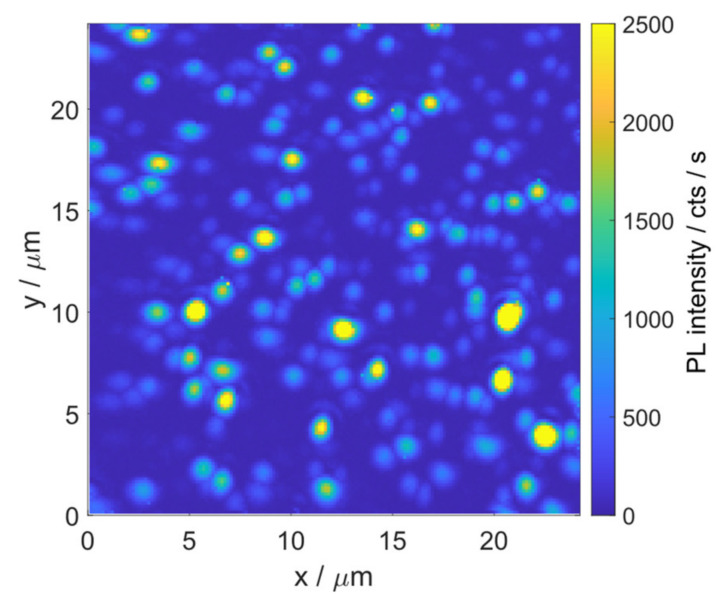
Exemplary µ-PL map of the photonic sample, measured at 4 K. The spectrometer ranges from 767 nm to 810 nm.

**Figure A5:**
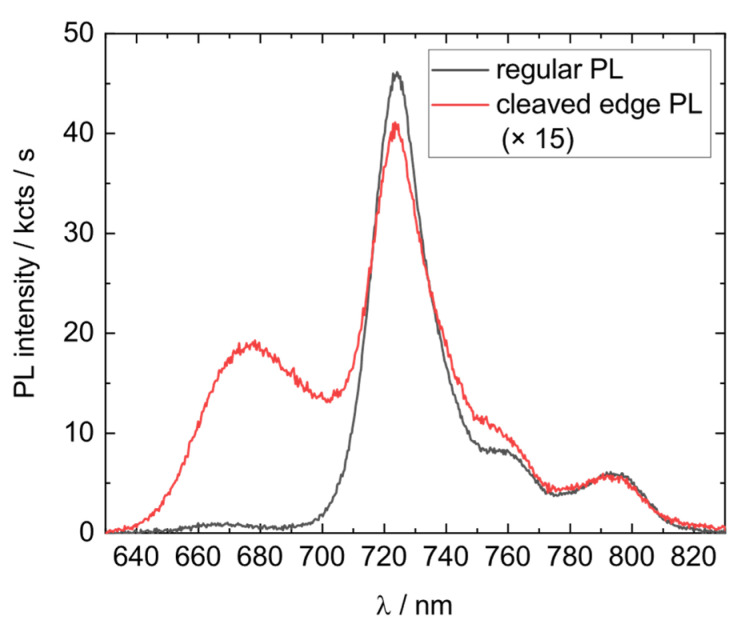
Comparison of regular and cleaved-edge ensemble PL. Intensity for cleaved-edge PL multiplied by a factor of 15 to match regular PL intensity.

**Figure A6:**
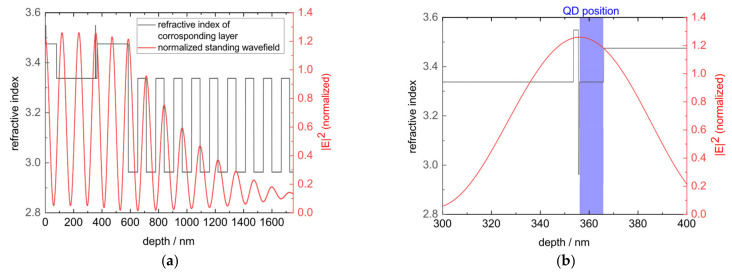
Simulated standing wave field of the photonic sample at the design wavelength and temperature (795 nm, 4 K) as an overview (**a**) and a more detailed view at the QD region in (**b**).

## Data Availability

The data presented in this study are available on reasonable request from the corresponding author.

## Appendix A. Layer Structure

**Table A1:** The grown layer structure with relevant breaks, substrate temperature, arsenic beam equivalent pressure (As-BEP), and intended doping concentration. * points to the step above.

Thickness/Time	Material/Operation	T_s_/°C	As−BEP/Torr	Intended Doping/cm^−3^
Surface				
5 nm	GaAs:C	600	9.6 × 10^−6^	8 × 10^18^
10 nm	Al_0.15_Ga_0.85_As:C	600	9.6 × 10^−6^	8 × 10^18^
65 nm	Al_0.15_Ga_0.85_As:C	600	9.6 × 10^−6^	2 × 10^18^
273.6 nm	Al_0.33_Ga_0.67_As	600	9.6 × 10^−6^	undoped
120 s	annealing GaAs annealing	635 −> 600	9.6 × 10^−6^	undoped
2 nm		635	9.6 × 10^−6^	undoped
60 s		635	9.6 × 10^−6^	undoped
60 s 0.37 nm/1.3 ML_eq_ 60 s	etching aluminum pump pump	635 635 635	9 × 10^−8^ −> 6 × 10^−8^ 9 × 10^−8^ *−> 9 × 10^−8^	undoped undoped undoped
180 s		620 −> 635	9.6 × 10^−6^−> *	undoped
30 s 10 nm	annealing Al_0.33_Ga_0.67_As	620 620	9.6 × 10^−6^ 9.6 × 10^−6^	undoped undoped
15 nm	Al_0.15_Ga_0.85_As	620	9.6 × 10^−6^	undoped
5 nm	Al_0.15_Ga_0.85_As	605	9.6 × 10^−6^	undoped
150 nm	Al_0.15_Ga_0.85_As:Si	620	9.6 × 10^−6^	2 × 10^18^
50 nm	Al_0.15_Ga_0.85_As	620	9.6 × 10^−6^	undoped
DBR: 10 × 67.08	AlAs	620	9.6 × 10^−6^	undoped
DBR: 10 × 57.54 nm	Al_0.33_Ga_0.67_As	620	9.6 × 10^−6^	undoped
SPS: 22 × 2.8 nm	GaAs	620	9.6 × 10^−6^	undoped
SPS: 22 × 2.8 nm	AlAs	620	9.6 × 10^−6^	undoped
Buffer: 10 × 10 nm	GaAs	620	9.6 × 10^−6^	undoped
625 µm GaAs (001) Substrate				

## Appendix B. Gradient Growth and Effective Etch Material

In this section, we describe how we achieved material and thickness gradients during epitaxy. As MBE effusion cells are mounted in a tilted nature in the growth chamber, the flux reaching the substrate always forms a parallax. This is unwanted for homogeneous-thickness layer growth and is usually counteracted by rotating the substrate during deposition. However, by stopping the rotation, it is possible to use the parallax for thickness variation on a single wafer, a so-called “gradient”. Applications for gradients in LDE are, for example, for variation of the hole filling or deposition of etching material.

In this work, the gradient technique was used to evaluate an estimation of the effective amount of etching material. In the photonic sample, the amount of Al used as the etching material corresponds to an equivalent of 1.3 ML_eq_. For our systematic study on the etching material, the amount was reduced until insufficient material for hole etching was reached, quantified by the absence of any QD-related PL signal. The wafer was oriented with the big-flat (bf) pointing towards the Al-cell. This results in a reduction of the Al deposited when moving away from the bf as is illustrated in Figure A1.

The PL maps of the samples are analyzed to find transitions from regions where GaAs QD emissions can be seen to regions without emission. The integrated PL intensity is plotted over the range of 750 nm to 830 nm for a central deposition of 0.5 ML_eq_ and 0.3 ML_eq_ in Figure A2a,b. The PL emission only occurs where enough Al is deposited to form large enough droplets to etch into the surface. The QD emission vanishes at a certain wafer position because there is not enough etch material for droplet formation, so no QDs can form. In the following, we refer to this border between the two regions as the “transition”. This transition has a convex form, which is caused by the effusion cell profile, with a small dip in the mid-wafer region, which is probably caused by an inhomogeneous temperature effect.

In Figure A3a,b, the corresponding Al equivalent to an ML thickness for 0.5 ML_eq_ and 0.3 ML_eq_ central thickness is simulated. The simulation is calibrated beforehand by quantum well growth and characterization, from which a position-dependent cell flux is calculated. It shows a typical convex evaporation cell profile.

By comparing simulation and PL emissions, the critical amount of aluminum needed for light-emitting LDE GaAs QDs can be determined. For the 0.3 ML_eq_ sample, the transition occurs from 0.345 to 0.392 ML_eq_, while for the 0.5 ML_eq_ sample, it ranges between 0.375 and 0.412 ML. The slightly higher amount of etching material needed in the 0.5 ML_eq_ sample is due to the transition being closer to the As-cell and the higher As-flux consuming some of the Al etch material by creating AlAs.

## Appendix C. Exemplary µ-PL Map

In Figure A4, an exemplary µ-PL map is plotted. It can be used to determine the QD density in the recorded wavelength interval. In order to also detect weak emitting QDs, the intensity scale was adjusted to lower values compared to Figure A4.

## Appendix D. Proof-of-Concept on Cleaved-Edge PL

In Figure A5, a cleaved-edge PL measurement is compared to a regular PL measurement on the non-photonic sample. It is noticeable that the whole spectrum looks qualitatively similar for wavelengths longer than 710 nm. Small changes can be seen in peak intensity, perhaps due to different absorption. A major difference is seen for emissions at wavelengths than shorter 710 nm, as shorter wavelength features are mostly absorbed by Al_15_GaAs layers in regular PL, but are well visible by cleaved-edge PL: The QDs at 760 and 797 nm do not show a noticeable difference, demonstrating that regular and cleaved-edge PL measurements are comparable, if no photonic heterostructure is involved. The difference in PL for samples with a photonic heterostructure (see main text) thus originates solely from the photonic structure.

## Appendix E. Standing-Wave Field of the Photonic Sample

Figure A6 hosts a simulation of the standing wave field in the photonic sample. While the extraction efficiency is equivalent to one minus the reflectivity, the emission enhancement scales with the standing wave field amplitude square is |E|^2^. The QD optically active region is close to the field maximum, causing additional enhancement.

## Appendix F. Fit-Parameters

Table A2 shows the parameters that were obtained by fitting several macro-PL spectra. The fits are used to illustrate the visible spectral change in Section 3.2 Figure 7. Values marked with * were constrained for physical consistency (approximately equidistant level-splitting, FWHM in plausible range), otherwise the fits would diverge. S- and P-states were unconstrained for all fits, except for Figure 7d (4.5 mW), where the position was fixed to the fit-value from the low power measurement.

**Table A2:** Fit parameters for the Gaussian-fits in Figure 7, including position, peak height, and FWHM. * marks constraint fit parameters.

Figure	Peak	Height/kcts/s	Position/nm	FWHM/meV
Figure 7b	S (1.2 mW) P (1.2 mW) D (1.2 mW)	14.9 ± 0.2 6.8 ± 0.2 1.1 ± 0.2	791.6 ± 0.2 778.7 ± 0.2 766.9 ± 1.4	21.7 ± 0.6 23 ± 2 20.3 *
Figure 7b	S (1.6 mW) P (1.6 mW) D (1.6 mW) F (1.6 mW)	25.9 ± 0.3 27.3 ± 0.2 11.7 * 2.2 *	792.2 ± 0.2 778.5 ± 0.1 766.3 ± 0.3 755.0 *	24.5 ± 0.5 23.8 ± 0.7 25.0 * 27 ± 7
Figure 7d	S (1.5 mW)	25.72 ± 0.07	801.0 ± 0.1	62.7 ± 0.3
Figure 7d	S (4.5 mW) P (4.5 mW)	54.5 ± 0.5 57.6 ± 0.2	801.0 * 780.5 ± 0.1	65.8 ± 0.4 59.9 ± 0.4
